# Assessing the Relationship Between Schizophrenia Symptom Severity and Insight Using the Frankfurt Complaint Scale (FBS) and “My thoughts and feelings” Questionnaire

**DOI:** 10.1192/j.eurpsy.2025.2228

**Published:** 2025-08-26

**Authors:** W. Chojna, S. Rusinek, M. Laskowska, M. Bezeg, D. Piłat, K. Krysta

**Affiliations:** 1 Department of Rehabilitation Psychiatry, Medical University of Silesia, Katowice, Poland

## Abstract

**Introduction:**

Schizophrenia remains a complex psychiatric disorder characterized by varying symptoms and levels of insight. The Frankfurt Complaint Scale (FBS) and the “My thoughts and feelings” questionnaire provide quantitative measures of symptom severity and insight, respectively. Previous literature has emphasized the importance of these tools in both clinical assessment and therapeutic planning.

**Objectives:**

Exploring the Relationship Between Schizophrenia Symptom Severity and Insight based on the Frankfurt Scale and the “My thoughts and feelings” Questionnaire.

**Methods:**

The study utilized data extracted from an Excel dataset comprising demographic information and specific scores from the FBS and the Insight questionnaire. The sample was divided into four subgroups based on their FBS scores, and the average Insight score for each subgroup was calculated.

**Results:**

The study found the following average Insight scores across FBS-defined subgroups: Mild symptoms: 9.67, Moderate symptoms: 8.25, Severe symptoms: 8.22, Very severe symptoms: 9.29. The correlation analysis revealed a weak and non-significant correlation between FBS scores and Insight scores (r = -0.017, p = 0.925). The demographic analysis showed a prevalence of male patients (n=20), with the most common age group being 36-45 (n=11). Most participants resided in large cities (n=16), with the highest educational attainment being a Bachelor’s/Master’s degree (n=12), and the majority were single (n=21). The study group displayed diverse demographic characteristics,

with a significant male predominance and a concentration in urban environments. This demographic distribution may influence the generalizability of the findings and provides a context for interpreting the varied experiences and perceptions of illness within the group.

**Conclusions:**

This study underscores the complexity of schizophrenia, where symptom severity does not straightforwardly correlate with insight into the illness. It highlights the importance of using a range of assessment tools to fully capture the multifaceted nature of patient experiences. Future studies should explore these relationships further to refine the tools used for assessments and to tailor intervention strategies effectively.

**Disclosure of Interest:**

None Declared

